# 10 Tips for Planning and Publishing Clinical Research

**DOI:** 10.1016/j.jacadv.2023.100647

**Published:** 2023-10-18

**Authors:** Verdoia Monica, Giuseppe Biondi-Zoccai, Velagapudi Poonam

**Affiliations:** a Nuovo Ospedale degli Infermi, ASL BI, Biella, Italy; b Department of Medical-Surgical Sciences and Biotechnologies, Sapienza University of Rome, Latina, Italy; c Mediterranea Cardiocentro, Napoli, Italy; d Department of Cardiology, University of Nebraska Medical Center, Omaha, Nebraska, USA

**Keywords:** research, statistics, virtual education

Scientific research is complementary to clinical activity and can be beneficial for both cardiovascular trainees and established cardiologists. Research forces clinicians to think about research methodologies, pathophysiological mechanisms of disease, and critically review outcomes. Research helps doctors to become better clinicians.

Although cardiology training programs and clinical programs often have journal clubs, grand rounds, and other activities to highlight research, less time is spent discussing how to conduct and publish research. Scientific societies have developed several educational initiatives dedicated to statistics and manuscript writing including formal courses, tutorials, and papers.^1^, ^2^, ^3^ Recently, there has been a growth of virtual and remote learning platforms including less formal social media educational activities.^4^ A growing number of options have become available for engaging with experts, statisticians, and journal editors, narrowing the gap between clinicians and the scientific community.^5^ Junior doctors, fellows, and early-career physicians are generally the most active promoters and beneficiaries of these initiatives. With the support of the American College of Cardiology Early Career Section, we discussed practical advice for planning research studies and preparing manuscripts with Dr Giuseppe Biondi-Zoccai (Editor-in-Chief, *Minerva Cardiology and Angiology*) and Dr Candice Silversides (Editor-in-Chief, *JACC: Advances*).^6^ Table 1 summarizes our take-home messages.

**Table 1:** Recommendations for Planning and Publishing Clinical Research

Clinical Research Tips	
Planning your study	• Ensure you have a clear protocol • Define key variables and outcomes • Distinguish between primary and secondary analyses • Be aware of risk of type I error • Consider sensitivity or exploratory analyses • Consider multidisciplinary collaborations when appropriate
Writing your manuscript	• Review the literature carefully • Ensure a clear structure to the manuscript • Use the recommended journal format • Addressing reviewers' comments considerately

Good studies start with careful planning and design, anticipating expected results as well as planning for potential obstacles.^7^ Study endpoints are very important and need to be very clearly considered and defined. The selection of the correct study variables can contribute to the success or failure of a research project. Several drugs and device trials have been ruined by inappropriate endpoint selection. This has been, for example, the case of cangrelor, which required 3 different trials and a subsequent meta-analysis^8^ to demonstrate its effectiveness, therefore delaying its routine use in clinical practice. In fact, the first trial, the CHAMPION PLATFORM trial,^9^ comparing cangrelor vs clopidogrel in more than 5,000 patients undergoing percutaneous coronary intervention, observed a nonsignificant trend of benefit with cangrelor (HR: 0.87; *P* = 0.17). The primary outcome of the study was death, myocardial infarction (MI), or revascularization within 48 hours of randomization. However, there was a problem with the definition of MI, especially in relation to periprocedural MI. When the definition was changed in the CHAMPION PHOENIX trial, the benefits of the new drug became evident, allowing its introduction into guidelines.^10^ Different priorities should be given to primary and ancillary analyses, especially when the latter represents exploratory or hypothesis-generating data not supported by statistical power.^11^ However, the inclusion of ancillary analyses in studies can provide additional value, paving the way for future investigations and evolving scientific processes. In addition, it is often helpful to work with statisticians early to ensure that statistical issues are considered early in the study design. Awareness of the potential sources of bias and systematic errors is crucial, as these sources of error can lead to false positive or false negative results. Furthermore, when writing the manuscript, it is important to include a detailed discussion of these potential biases.^12^ Multidisciplinary and multisite collaborations can be useful and allow for different viewpoints and approaches, increase the size of the study population, and help diversity the population being studied.

Once the study is complete, manuscript preparation begins. Before writing the manuscript, ensure that you are updated on the existing literature. When writing the introduction, ensure that you explain what is known about the topic, what is missing, and how your study helps to fill the knowledge gap. Even excellent studies may have difficulty getting published if they are not well written.^13^^,^^14^ While you may wish to find a captivating title, ensure that it describes the study clearly. Identifying the most appropriate journal for submission is very important and can save time and prevent repeated rejections. Respect the journal style and formatting, as this helps editors and reviewers appraise your manuscript more efficiently. Finally, ensure you carefully review the manuscript before submission. Reviews, if done well, can help to improve a manuscript, so use them to your advantage. If you are submitting a revised manuscript, address all the reviewers’ comments with the aim of improving the manuscript.

In conclusion, following simple rules and seeking advice from more experienced colleagues and field experts is the key to success in conducting clinical research, from creating the study protocol to publishing final results. This long process requires motivation as well as the 3 “P”s for success in academics: persistence, planning, and picking the right opportunity.

## Funding support and author disclosures

Dr Verdoia has received speaking honoraria from BBraun and Sanofi. Dr Biondi-Zoccai has consulted for Amarin, Balmed, Cardionovum, Crannmedical, Endocore Lab, Eukon, Guidotti, Innovheart, Meditrial, Microport, Opsens Medical, Terumo, and Translumina. Dr Velagapudi has received speaking fees from Opsens, Medtronic, Shockwave, and Abiomed; and has participated on advisory boards for Abiomed and Sanofi.
